# Supplementary material for: Implementation research on management of sick young infants with possible serious bacterial infection when referral is not possible in Jimma Zone, Ethiopia: Challenges and solutions
Source: PLoS One. 2021 Aug 9;16(8):e0255210. doi: 10.1371/journal.pone.0255210 (PMC8351942; doi:10.1371/journal.pone.0255210)
Supplement: Attachment — Submitted filename: Response to Reviewers.docx [file pone.0255210.s001.docx]

**Point by point response to the points raised by the academic editor and reviewer(s)**

Thanking the academic editor and reviewer(s) for their comments and suggestions, we hereby provide the point by point responses to the comments and suggestions.

**Review Comments to the Author**

**Reviewer #2:** I appreciate the rather extensive description of the Ethiopian HEP and its components, but feel that this time it has become almost too extensive. Also the text contains a few wordings that indicate an evaluation of the HEP without giving any reference to a scientific analysis of the program. Thus, I would like the authors to slim down the text describing HEP, but keep the essentials (that is a great help to understand the paper) and avoid value loaded statements. Otherwise I am fully satisfied with the revision.

**Our response:** We have included the respective references at the respective place. We have also modified the texts accordingly (panel 1).
